# Mesenchymal stromal cells (MSCs) and their exosome in acute liver failure (ALF): a comprehensive review

**DOI:** 10.1186/s13287-022-02825-z

**Published:** 2022-05-08

**Authors:** Samin Shokravi, Vitaliy Borisov, Burhan Abdullah Zaman, Firoozeh Niazvand, Raheleh Hazrati, Meysam Mohammadi Khah, Lakshmi Thangavelu, Sima Marzban, Armin Sohrabi, Amir Zamani

**Affiliations:** 1 Department of Research and Academic Affairs, Larkin Community Hospital, Miami, FL USA; 2 I.M. Sechenov First Moscow State Medical University (Sechenov University), Moscow, Russian Federation; 3 Basic Sciences Department, College of Pharmacy, University of Duhok, Duhok, Kurdistan Region Iraq; 4 School of Medicine, Abadan University of Medical Sciences, Abadan, Iran; 5 Department of Medicinal Chemistry, Pharmacy Faculty, Tabriz University of Medical Sciences, Tabriz, Iran; 6 Department of Oral and Maxillofacial Surgery, School of Dentistry, Shahid Beheshti University of Medical Sciences, Tehran, Iran; 7 Department of Pharmacology, Saveetha Dental College, Saveetha Institute of Medical and Technical Science, Saveetha University, Chennai, India; 8 Student Research Committee, Tabriz University of Medical Sciences, Tabriz, Iran; 9 Immunology Research Center, Tabriz University of Medical Sciences, Tabriz, Iran; 10 Stem Cell Research Center, Tabriz University of Medical Sciences, Tabriz, Iran

**Keywords:** Mesenchymal stromal cell (MSCs), Exosome, Acute liver failure (ALF), Immunomodulation, Hepatocyte

## Abstract

Recently, mesenchymal stromal cells (MSCs) and their derivative exosome have become a promising approach in the context of liver diseases therapy, in particular, acute liver failure (ALF). In addition to their differentiation into hepatocytes in vivo, which is partially involved in liver regeneration, MSCs support liver regeneration as a result of their appreciated competencies, such as antiapoptotic, immunomodulatory, antifibrotic, and also antioxidant attributes. Further, MSCs-secreted molecules inspire hepatocyte proliferation in vivo, facilitating damaged tissue recovery in ALF. Given these properties, various MSCs-based approaches have evolved and resulted in encouraging outcomes in ALF animal models and also displayed safety and also modest efficacy in human studies, providing a new avenue for ALF therapy. Irrespective of MSCs-derived exosome, MSCs-based strategies in ALF include administration of native MSCs, genetically modified MSCs, pretreated MSCs, MSCs delivery using biomaterials, and also MSCs in combination with and other therapeutic molecules or modalities. Herein, we will deliver an overview regarding the therapeutic effects of the MSCs and their exosomes in ALF. As well, we will discuss recent progress in preclinical and clinical studies and current challenges in MSCs-based therapies in ALF, with a special focus on in vivo reports.

## Introduction

Acute liver failure (ALF) is characterized by the occurrence of coagulopathy (international normalized ratio [INR] > 1.5) and any level of encephalopathy emerging 24 weeks following the occurrence of the first symptoms in patients who have no history of previous liver disease [1]. The timing and the level of clinical presentation can be classified into three types: hyperacute, acute, and subacute [2]. Hyperacute and acute types involve fulminant hepatic failure, while the subacute type is also named subfulminant [3]. Interestingly, the mortality rate among the patients whose hepatic encephalopathy appears 8 weeks after the onset of symptoms (fulminant hepatic failure) is lower than the patients with a more gradually evolving course [4]. Multiorgan failure (MOF) has proved to be the main cause of death (> 50%) from ALF, while intracranial hypertension (ICH) and infection are the other main causes of mortality in this patient population [5].

During last two decades, a diversity of stem cells, such as pluripotent stem cells (PSCs), mesenchymal stromal cells (MSCs), hepatic progenitor cells (HPCs), and hematopoietic stem cells (HSCs), has been used to treat liver diseases [6–8]. However, MSCs are the most common type used in research, since they pose no ethical challenges and can be obtained easily [9, 10]. Results show that MSCs have the capability of differentiating more than once; moreover, they can self-renew. They can differentiate into an array of cell lineages, including hepatocyte-like cells (HLCs) [11]. MSCs are also characterized by other properties, such as anti-inflammatory, antiapoptosis, antifibrotic, antioxidant, blood vessel formation, improvement of tissue repair, and growth factor secretion [12, 13]. Despite many preclinical and clinical investigations on MSCs used in treating ALF, it is still unknown what mechanism contributes to the therapeutic effect of MSCs. Besides, MSCs-exosomes have caught the attention of many researchers as a new cell-free method regarding the regeneration of the liver [14, 15]. They have dissipated the worries concerning the direct application of MSC (e.g., immunogenicity and tumor formation [16]. Such exosomes encompass high frequencies of cytoplasmic and membrane proteins, including enzymes, transcription factors, lipids, ECM proteins. They also include nucleic acids, such as mitochondrial DNA (mtDNA), single-stranded DNA (ssDNA), double-stranded DNA (dsDNA), messenger RNA (mRNA), and microRNA (miRNA) [17]. The size of exosomes varies from 30 to 150 nm, and they can be transferred to other cells to do their functions. As a highly controlled process, the generation of exosome from the other organisms similar to themselves is comprised of three main steps: (1) formation of endocytic vesicles by the folding of the exterior area of the plasma membrane, (2) formation of multivesicular bodies (MVBs) by inward budding of the endosomal membrane, and (3) incorporation of established MVBs with the plasma membrane and secretion of the vesicular contents, called exosomes [14, 18]. Exosomes elicit antioxidant effects and motivate target cells to trigger downstream signals. Moreover, they convey genetic material to target cells, leading to the suppression of inflammation and apoptosis. [19, 20].

This review aims to give an overview of the present knowledge to elucidate mechanisms used by MSCs to underlie liver restoration in ALF. Another aim is to present a discussion of new developments in preclinical and clinical investigations on MSCs therapy in liver-associated diseases, with a particular focus on ALF.

## Pathophysiology of ALF

Acetaminophen (APAP) has proved to be the main cause of ALF [21]. The following people are highly likely to experience ALF stimulated by APAP: alcoholic people who use APAP; people who suffer from malnutrition; or people who take medications that are believed to induce CYP450 enzymes (e.g., phenytoin, carbamazepine, or rifampin). Results of a study on 308 patients with severe liver disorder in the USA revealed APAP as the main cause of ALF in 40% of patients [22]. The other causes detected were as follows in the increasing order of prevalence:

- Malignancy (1%)
- Budd-Chiari Syndrome (2%)
- Pregnancy (2%)
- Wilson disease (3%)
- Hepatitis A virus infection (4%)
- Autoimmune hepatitis (4%)
- Ischemic hepatitis (6%),
- Hepatitis B virus infection (6%)
- Idiosyncratic drug-induced liver injury (13%)

The causes of 17 percent of cases were not known [4].

Based on results, it is possible to categorize the ALF pathophysiology into two groups: pathophysiologies of liver problems involving a specific cause and pathophysiology concerning the appearance of secondary multiorgan failure (MOF) [23]. With regard to the pathophysiology of liver disorders, the results show that APAP toxicity is the main cause [24]. Secondary MOF often derives from the primary extensive pro-inflammatory effect, which leads to a pervasive inflammatory effect syndrome. Then, a deregulated anti-inflammatory response ensues [25, 26].

It is not clear what mechanism causes the ongoing death of tissue when there is no ongoing injury. Oxidative stress results in the formation of reactive oxygen species (ROS). This, in turn, activates c-Jun N-terminal kinase (JNK) through a series of events [27]. Such activation may support disruption of normal mitochondrial function**,** which inspires more hepatocyte necrosis and damage associated molecular patterns (DAMPs) [28, 29]. DAMPs bring about the activation of hepatic macrophages, resulting in the formation of the inflammasome [30, 31]. Concisely, as complexes characterized by multiple proteins, inflammasomes receive the intracellular danger signals through NOD-like receptors (NLRs) [32]. The inflammasome effectively regulates the inflammatory response by eliciting a response to low-threshold signals. Toll-like receptors (TLRs) induction by DAMPs leads to the inflammasome activation, supporting the subsequent activation of caspase-1 and IL-1β secretion [33, 34]. Researchers have identified the characteristics of the NLR family pyrin domain containing 3 (NLRP3) inflammasome belonging to the inflammasome family. NLRP3 inflammasome has three potential activation pathways: (1) ATP signal which occurs outside a cell, leading to potassium efflux and pannexin recruitment; (2) incorporation of crystalized cholesterol, uric acid or amyloid with lysosomal dysfunction after the ingestion and elimination of these particles; and (3) activation by reactive oxygen species (ROS) [33, 35, 36]. Investigations have examined the activation of inflammasome in APAP-induced ALF by a special focus on the contribution of the inflammasome to acute liver disorder [37]. It appears that DAMPs are released from necrotic hepatocytes and sinusoidal endothelial cells, leading to the activation of the inflammasome in the manner mentioned above.

## The rationality of MSCs therapy in ALF

**Fig. 1:**
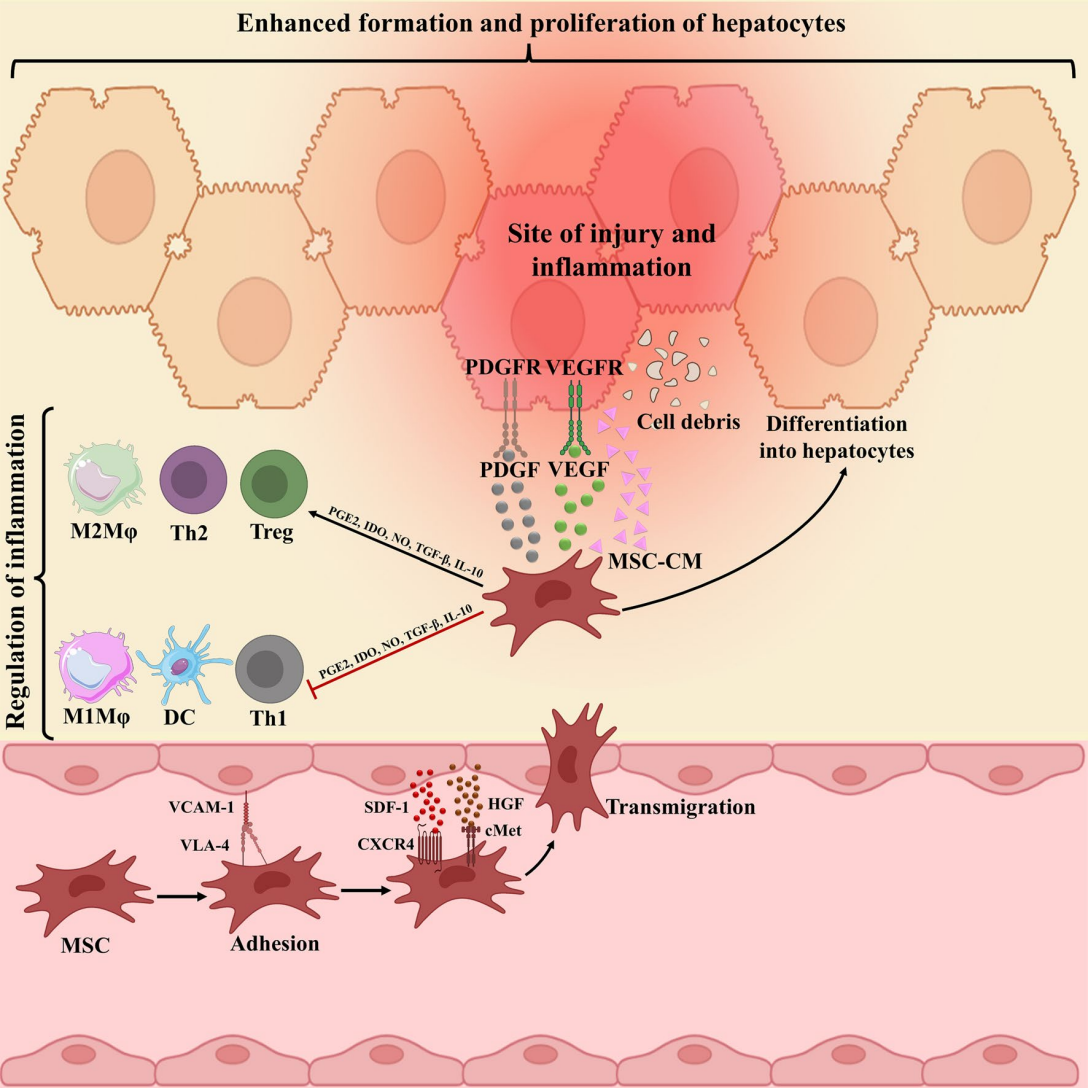
Underlying mechanism complicated in mesenchymal stromal cells (MSCs) migration to damaged liver tissue. The connections between CXCR4 and SDF-1ɑ, c-Met and HGF, and finally VLA-4 and VCAM-1 underlie cell to cell interaction between endothelial cells (ECs) and MSCs, which, in turn, facilitate MSCs migration to damaged liver tissue. Then MSCs secrete anti-inflammatory molecules, such as PGE2, IDO, TGF-β, IL-10, and NO to down-regulate inflammation. These molecules prompt the change of inflammatory to proliferating phase largely defined by the secretion of PDGF and VEGF, sustaining hepatocyte formation and proliferation

MSCs migration to damage tissue by interaction with several receptors and molecules, and thereby inducing liver recovery by various mechanisms has been proved (Fig. 1). Although the mechanisms of MSCs transplantation are still not entirely understood, a growing body of proof has indicated that their immunomodulation, differentiation, and antifibrotic capabilities play central roles in liver repair. Among them, anti-inflammatory potential of MSCs play most critical role. Although there is no clear evidence indicating the MSCs in vivo differentiation into hepatoid cells post-transplantation, MSCs can be differentiated into hepatocyte-like cells (HLCs) in vitro and then be infused. Of course, this process is time-consuming process with insufficient established HLCs, thereby hindering its therapeutic utility in clinic. However, there is some evidence indicating that replacing fetal bovine serum (FBS) with polyvinyl alcohol (PVA) might lead to improved differentiation ability [38]. In vivo, as only a small number of intravenously injected cells reach the liver, MSCs-mediated favored effects mainly depend on the secreted molecules rather than their direct effects or differentiation into hepatocytes post-transplantation [39].

### MSC anti-inflammatory properties

The hepatocyte loss is the first symptom and mechanism contributing to acute liver damage. It is still unclear what causes ongoing necrosis when there is no injury. ROS are produced in response to oxidative stress. This, in turn, activates c-Jun N-terminal kinase (JNK) through a series of events, resulting in mitochondrial dysfunctions. These events lead to a higher level of hepatocyte necrosis, as well as the expansion of DAMPs [40]. DAMPs stimulate the activation of hepatic macrophages and the formation of the inflammasome [41]. In the next stage, the release of pro-inflammatory cytokines eases the recruitment of a larger number of immune cells to the inflammation area, and so advances hepatocyte cell necrosis.

The majority of past investigations have indicated that MSCs play a therapeutic role in the treatment of liver dysfunction by releasing trophic factors and the factors modulating the immune system [42]. Although the role of MSCs in modulating the immune system is unclear, they might control the immune cells through the secretion of soluble factors and the contacts between cells. The regulation of adaptive and innate immune responses by MSCs is exerted by inhibiting T cells and dendritic cells (DCs), which leads to a reduction in the activation and growth of B cells [43, 44]. This, in turn, enhances the formation of regulatory T (Treg) cells and prevents the growth and toxicity of natural killer (NK) cells induced by the chemotherapeutic molecules [45]. Also, transforming growth factor-beta (TGF-β) and interleukin 10 (IL-10) as crucial factors in the regulation of a large number of inflammatory cells [46, 47]. Studies revealed a significant increase in the amounts of TGF-β and IL-10 in serum following the injection of UC-MSCs, but a significant decrease in the amounts of IL-6, tumor necrosis factor-alpha (TNF-α), and cytotoxic T lymphocytes (CTLs) was seen in peripheral blood [48]. This led to the restoration of liver function, as well as a reduction in the development of disease and the level of mortality. Furthermore, transient T cell apoptosis can be induced by BM-MSCs through the Fas ligand (FasL)-dependent pathway [49]. Then, macrophages are stimulated by apoptotic T cells to form high amounts of TGF-β, resulting in the up-regulation of Treg cells to trigger immune tolerance. MSCs can prevent cytotoxic CTLs and NK cells through the contact between cells and paracrine factors, including indoleamine 2,3-dioxygenase (IDO), TGF-β, and prostaglandin E2 (PGE2) [50, 51]. Of course, TGF-β acts as a two-edged sword. It can weaken the immune system and thereby suppress liver inflammation [49]; on the other hand, it can increase liver fibrosis [52]. MSCs, in fact, can act as an immunomodulatory agent in reducing the inflammation of the body through up-regulating anti-inflammatory Treg cells and decreasing T helper 1 (Th1) and Th17 cells in ALF [53]. Moreover, the inflammation after MSC transplantation can be indirectly stimulated by up-regulating M2-type macrophages, leading to the secretion of a variety of anti-inflammatory factors, such as chemokine ligand 1 (CCL-1) and IL-10, up-regulation Th2, and Treg cells [54]. Also, MSCs play an important role in the reduction of B-cell growth through contact between cells and the secretion of soluble factors [55]. Finally, MSC transplantation can play an effective role in mitigating liver damage in ALF by decreasing the number and activity of neutrophils in both peripheral blood and the liver.

### MSCs differentiate into HLCs

MSCs are characterized by their ability to proliferate and differentiate in vitro. For the first time, Friedenstein et al. procured MSCs in 1968 from the bone marrow (BM) [56, 57]. After that, MSCs obtained from multiple sources, making them an excellent supply of multipotent cells for treatment of liver dysfunctions. A variety of methods are used to differentiate MSCs into HLCs [58, 59]. Studies show that multiple signals contribute to the regulation of the cells’ behavior in a cooperative manner. Such signals are usually triggered by extracellular matrix (ECM), growth factors, and even juxtacrine signals [60]. Each one of the organs, as well as the developmental stage, is characterized by a specific regulated timing and distribution pattern of signals. As a result, achieving better results in the case of in vitro cultures requires establishing a type of environment that resembles the local environment. Based on the research previously done, it is possible to differentiate MSCs obtained from various sources into hepatocytes in the case of both mice and humans through the implementation of a variety of protocols and methods in vitro [61, 62].

Hepatic differentiation protocol is known to be the most frequently used method for hepatic differentiation, which benefits from Iscove's Modified Dulbecco's Medium (IMDM), as well as cytokine cocktail. Epidermal growth factor (EGF) or fibroblast growth factors (FGF) trigger the MSCs to differentiate into endodermal cells during the early induction stage. EGF prompts the MSCs to proliferate by interfaces with EGF receptor (EGFR) [63]. Besides, FGF is a member of a bigger family that is comprised of seven polypeptides with similar characteristics [64]. This family plays an essential role in the primary stage of endodermal patterning [65]. In particular, FGF-4 and basic FGF (bFGF) are commonly used. Like EGF, FGF influences the growth rate of MSCs [66].

Generally, the differentiation of cells is stimulated by adding FGF, HGF, nicotinamide (NTA), and also insulin-transferrin-selenium (ITS) into cultures [67]. As a mesenchymal origin pleiotropic cytokine, hepatocyte growth factor (HGF) contributes to adjustment of growth, differentiation, and chemotactic migration of MSCs [68]. MSCs’ exposure to HGF for a short time causes the activation of c-Met receptors along with its downstream agents such as extracellular signal-regulated protein kinase (ERK)1/2, p38, mitogen-activated protein kinases (MAPKs), and phosphoinositide 3-kinase (PI3K) /Akt [69, 70]. MSCs’ exposure to HGF for a long time will make changes in cytoskeletal arrangement; moreover, it results in the migration of cells and a notable decrease in proliferation. In addition, ITS and NTA promote the growth and survival of primary hepatocytes [71].

Despite the fact that MSCs can differentiate in culture through induction, the organ-specific microenvironment is the best technique, enabling MSCs differentiation into a certain cell type. The ability to express hepatocyte-specific genes is one of the specific characteristics of hepatic-differentiated cells, which can be affected by microenvironmental features [72]. Reports display that in the case of humans, the differentiation of the MSCs obtained from umbilical cord (UC) into HLCs occurs more quickly in the fibrotic liver microenvironment [73].

Other studies also show that the differentiation of MSCs into functional hepatocytes does not occur directly; rather, these cells initially differentiate into biliary epithelial cells (BEC)-like cells, followed by differentiation into HLCs [74]. However, according to the results of other investigations, MSCs transdifferentiation infrequently occurs after MSC infusion in animal models [75]. MSCs obtained from menstrual blood, for instance, turned out to prevent hepatic satellite cells (HSCs) activation and resultant liver fibrosis, leading to the improvement of liver function. Yet, very few transplanted MSCs differentiated into functional HLCs in vivo [76]. These results demonstrate that the therapeutic impact of MSCs is mediated mainly by indirect paracrine signaling.

### MSCs antifibrotic properties

Thanks to their antifibrotic and immunosuppressive properties, MSCs play an important role in the treatment of fibrosis [77]. Also, fibrosis in not a common pathological signs of ALF; long-term liver damage mainly results in fibrosis. MSC transplantation could attenuate liver fibrosis by down-regulation of TGF-β1, Smad2, collagen type I, and smooth muscle alpha-actin (αSMA), reducing liver fibrosis regions in vivo [78]. Besides, BM-MSCs decreased hepatic collagen distribution by impairing the TGF-β/Smad signaling pathway in a cirrhosis rat models [79]. MSCs also ameliorated hepatic microvascular dysfunction and portal hypertension, which contribute to complications defining clinical decompensating [80]. Further, the expression of matrix metalloproteinase (MMP)-2, -9, -13, and -14 can be up-regulated by MSCs [81], which, in turn, attenuates liver fibrosis through degrading extracellular matrix (ECM) [82]. MSCs reinforce this effect by the down-regulation of the tissue inhibitors of matrix metalloproteinases (TIMPs). Importantly, there is an association between the balanced levels of MMPs and TIMP and fibrosis resolution [83]. Moreover, MSCs have both direct and indirect roles in inhibiting the activation and growth of hepatic satellite cells (HSCs) and thereby could inhibit collagen synthesis [84]. The direct interactional relationship between MSCs and HSCs helps to inhibit HSC proliferation by stimulating G0/G1 cell-cycle arrest. This is done by inhibiting the phosphorylation of ERK1/2 [85]. On the other hand, MSCs contain substantial levels of milk fat globule-EGF factor 8 (MFGE8). The MFGE8 reduces expression levels of TGF-β1 receptor on HSCs, thus strikingly fences primary human HSCs activation [86]. In co-culture conditions, MSCs also mainly impair α-smooth muscle actin (α-SMA) expression of HSCs, which is mediated, in part, by the activation of the Notch pathway [87]. The indirect secretion of some pivotal factors (IL-10, HGF, TGF-β, and TNF-α) by MSCs averts the growth of HSCs and reduces the formation of collagen. In contrast, HGF and NGF enhance the apoptosis of HSCs [88, 89].

### MSCs antioxidant properties

One of the events deriving from ROS is oxidative stress, which is known as a common driver in creating damage to the liver. Some of these damages include the liver failure, liver fibrosis, liver cirrhosis, viral hepatitis, and also hepatocellular carcinoma (HCC) [90, 91]. The results of some investigations have revealed that MSCs play a strong mediatory antioxidant role in different animal models [92, 93]. Oxidative liver injury is mostly caused by thioacetamide (TAA) or carbon tetrachloride (CCl4) as the most commonly used toxins. These types of toxins give rise to hepatocyte dysfunction through the peroxidation of lipid and proteins alkylation, nucleic acids, and lipids [94, 95], resulting in the inflammatory response, hepatocellular injury, and liver fibrosis. Cell signaling and homeostasis require a low level of physiologic ROS formed by the mitochondrial respiration. MSCs have proved to have the capability of mitigating oxidative stress simulated by CCl4 and TAA in vivo [96, 97]. Through enhancing the superoxide dismutase (SOD) expression and antioxidant response elements (AREs) stimulation, MSCs boost antioxidant and cytoprotective performance, leading to a reduction in hepatocyte apoptosis [98, 99]. Due to their antioxidant role along with their role in modulating the immune systems, MSCs can be very useful in developing therapies for liver injuries.

## The importance of MSCs-exosome as cell-free approach in ALF

Exosome is a main subtype of extracellular vesicles (EVs) with a diameter in the range of 40–150 nm. Exosome are mainly produced by a diversity of human cells, such as stem/stromal cells, immune cells, and tumor cells [100]. They include several biological components, more importantly, miRNAs, proteins, lipids and mRNAs, as cargo [101, 102]. The production and secretion procedure of exosome consists of three chief steps: (1) creation of endocytic vesicles through invagination of the plasma membrane, (2) creation of multivesicular bodies (MVBs) upon endosomal membranes’ inward budding, and (3) incorporation of created MVBs with the plasma membrane and releases of the vesicular contents termed exosomes [14, 18]. Then, the contents of exosomes are transferred to the recipient cells, and thereby modify physiological cells [15]. As a result of their great capabilities to elicit hepatoprotective effect, exosomes are recently been considered as a rational therapeutic option for liver failure, thereby circumventing comprehensions concerning stromal cells’ direct transplantation [103, 104]. They are smaller and less complex compared with parent cells, and thereby easier to produce and store. Also, they exhibit no risk of tumor formation. Importantly, exosomes are less immunogenic than their parent cells due to their lower membrane-bound proteins. Recently, UC-MSCs-derived glutathione peroxidase1 (GPX1) enriched exosome showed the capacity to compromise oxidative stress as well as apoptosis in the hepatocyte, stimulating hepatoprotective effect in ALF rodent models [105]. Also, MSCs-derived exosome potently reduced inflammatory response in ALF animal models by impairment of IL-6-mediated signaling axis [106] and also down-regulation of NLRP3 pathway [107]. However, further studies are strongly needed to entirely elucidate how MSCs-derived exosomes exert their hepatoprotective influences in vivo.

## MSCs in ALF (animal studies)

### Native MSCs

MSCs-based treatments have shown huge potential for regenerating the liver and repairing its injury, which resulted from several liver disorders (Tables 1, 2). In vivo, MSCs can migrate to damaged tissues and constrain the production of pro-inflammatory molecules (e.g., IL-1, IL-6, and TNF-ɑ) and ultimately potentiate liver cells growth. As described, the chief mechanism behind the MSCs-mediated positive effects is their immunoregulatory potential rather than direct differentiation into haptoid cells. These cells efficiently hinder the activation of both innate and adaptive immune system cells, such as neutrophils, macrophages, NK cells, DCs, monocytes, and also lymphocytes. Studies in liver failure animal models revealed that MSCs could transdifferentiate into albumin-expressing HLCs, and also may support normal hepatocytes proliferation in vivo upon fusion with them [108]. Findings have outlined the important roles of SDF-1/CXCR4 axis to ease MSCs migration to damaged tissue, sustaining liver rescue in ALF [108]. As well, injection of MSCs-derived hepatocyte into mice with liver failure ameliorated liver function, as evidenced by analysis of serum profile as well as biochemical factors rates [109]. Notably, the serum levels of TGF-β1 and IL-10 in transplanted animals were more prominent than in control animals [109]. Other studies displayed that pyroptosis, a unique shape of programmed cell death induced by penetrating inflammatory reaction, was suppressed by MSCs therapy in ALF preclinical model [110]. Accordingly, MSCs administration caused liver repair in C57BL/6 mice by up-regulation of IL-10 and concomitantly suppression of NLRP3 [110]. Given that NLRP3 inflammasome elicits liver failure through induction of procaspase-1 and pro-IL-1 β accompanied with the adjustment of downstream CD40-CD40L signaling, its inhibition as elicited by MSCs can enable liver recovery in ALF [111]. Besides, the study of the soluble factor produced by MSCs and their potent desired impacts in a rat model of ALF revealed that IL-10, which mainly is secreted by MSCs, has a central role in ALF recovery post-transplantation [112]. It was found that phosphorylated STAT3 diminished upon IL-10 injection and conversely STAT3 suppression abrogated IL-10-induced effects in vivo, reflecting the eminent role of STAT3 signaling in exerting IL-10-induced anti-inflammatory influences [112]. In addition to the IL-10, MSC-produced PGE2 could constrain apoptosis and simultaneously augment hepatocyte proliferation, thereby decreasing ALF [113]. In fact, PGE2 stimulated YAP activation and then activated YAP suppressed phosphatase and tensin homolog (PTEN) and consequently up-regulated mammalian target of rapamycin (mTOR), a foremost controller of cell growth. This axis in turn protected versus ALF through increasing hepatocyte proliferation [113]. Furthermore, there is clear evidence signifying that MSCs could modify phenotype and function of macrophages, adjust DCs either differentiation or maturation, and impede the T cell activities by the production of tumor necrosis factor-alpha-stimulated gene 6 (TSG-6) in ALF models [114]. TSG-6 mainly averts the inflammatory response as a result of suppressing P38 and JNK signaling axes, providing a suitable milieu for ALF rescue upon MSCs transplantation [115]. MSCs also can induce their favored influences by heme oxygenase (HO) 1, a rate-limiting enzyme in heme metabolism, which is noted as an effective antioxidative and cytoprotective molecule. Recently, it was proven that MSCs administration gave rise to HO-1 up-regulation, whereas suppressing HO-1 impaired MSCs-induced desired effects and also hepatocyte autophagy [116]. These favored effects upon MSCs therapy were most probably caused by PI3K/Akt signaling pathway-induced HO-1 up-regulation [116]. Also, Zhang et al. found that systemic administration of BM-MSCs into the ALF rat model attenuated ALF by up-regulation of the HO-1 expression and subsequent attenuation in neutrophil infiltration and activation [117]. This event finally reduced hepatocyte apoptosis and also improve their proliferation, culminating liver recovery. Similarly, the pivotal role of neutrophils in ALF pathogenesis has been clarified by other reports [118]. In the D-galactosamine-induced ALF animal model, the great number of neutrophils aggregated in the liver tissue along with promoted myeloperoxidase (MPO) activity and enhanced alanine aminotransferase (ALT) and aspartate aminotransferase (AST) serum levels are mainly detected [118]. Nonetheless, injection of BM-MSCs brought about functional recovery, which was documented by reduced ALT and AST levels and also improved survival rate in the treatment group compared with the control group (50% vs 12.5%). Notably, the intervention led to a robust decrease in the population of neutrophils in the liver, MPO function, and also the expression of pro-inflammatory factors, including TNF-α, IL-1β, interferon gamma (IFNγ) and CXC chemokine ligands 1/2 (CXCL1/2) [118]. In addition, in a monkey model of ALF, systemic administration of the MSCs derived from another source, unbiblical cord (UC), reduced hepatic aggregation and maturation of circulating monocytes and their IL-6 releases, resulted in prolonged survival [119]. UC-MSCs also could induce a reduction in ALF by provoking the endogenous liver regeneration in association with suppression of liver cell apoptosis by up-regulating HGF/c-Met signaling axis [120] or down-regulation of Notch and STAT1/STAT3 signaling [121]. The positive influences of MSC therapy on hepatocyte proliferation also may arise from activation of AKT/ glycogen synthase kinase 3 beta (GSK-3β)/β-catenin pathway and enhancement in glucose metabolism leading to improved survival rate in ALF animal model [122]. Interestingly, intraportal injection of MSCs showed superiority over hepatic intra-arterial, intravenous, and intrahepatic injection in terms of liver recovery rate in swine with ALF. Notably, the liver recovery might be attributable to down-regulation of caspase-3, up-regulation of apoptosis inhibitor survivin as well as activation of AKT and ERK axes [123]. On the other hand, another study revealed that systemic infusion of MSCs was more effective than the intraperitoneal (IP) injection to support liver recovery because of the more significant increase in expression levels of growth factor vascular endothelium growth factor (VEGF) [124]. Also, compared with BM-MSC, adipose tissue (AT)-derived MSCs displayed higher therapeutic capacities, as defined by estimation of ALT and AST levels post-transplantation in ALF murine model [125].

**Table 1:** Direct administration of native mesenchymal stromal cells (MSCs) in liver failure preclinical models, especially acute liver failure (ALF)

Sources	Model	Result (ref)
Placenta	Rat	Migration to damaged site and induction of immunomodulatory effects by secreting paracrine factors in ALF [193]
Bone marrow	Rat	Systemic administration of MSCs reduced ALT, AST, and bilirubin levels [124]
Bone marrow	Rat	Reducing ALF, improving glucose metabolism and survival, and also stimulation of the hepatocyte proliferation by activating AKT/GSK-3β/β-catenin pathway [122]
Adipose tissue	Rat	Normalization of amino acids, sphingolipids, and glycerophospholipids in the liver and blood along with attenuation hepatocyte apoptosis and conversely promoting their proliferation rate [194]
Placenta	Rat	Stimulation of liver repair through the antifibrotic and autophagic mechanisms [149]
Umbilical cord	Monkey	Inhibition of the activity of IL-6 producing monocyte, amelioration of the liver histology, and also animal survival [119]
Adipose tissue	Rat	Suppression of the secondary complications of liver failure [195]
Bone marrow	Porcine	Improving the liver function homeostasis, attenuation of reactive oxygen species (ROS) following efficient homing, and also differentiation into hepatocytes [196]
Bone marrow	Rat	Amelioration of mitochondrial activities and normalization of lipid metabolism upon modifying the mTOR pathway [197]
Umbilical cord	Rat	Provoking the endogenous liver regeneration, hindrance of hepatocyte apoptosis by up-regulated c-Met in hepatocyte [120]
Bone marrow	Rat	Potentiating of MSCs-elicited liver regeneration following the abrogation of autophagy in MSCs [198]
Bone marrow	Rat	Amelioration of ALF by up-regulation of the heme oxygenase 1 (HO-1) expression, which resulted in inspiring the autophagy process through PI3K/AKT signaling axis [116]
Bone marrow	Mice	Enhancing MSCs competencies to stimulate liver recovery following transdifferentiation as well as fusion with hepatocytes by SDF-1/CXCR4 axis [199]
Bone marrow	Mice	Reducing ALF by IL-10 produced by MSCs, which ultimately inhibits pyroptosis [110]
Bone marrow	Mice	MSCs derived from adipose tissue showed superiority over MSCs isolated from bone marrow in ALF [125]
Bone marrow	Mice	Improvement of hepatocyte mediated by PGE2 released by MSCs, ameliorating ALF [113]
Wharton’s jelly	Mice	Restoration of hepatotoxicity by WJ-MSC [200]
Bone marrow	Swine	Averting ALF upon stimulation of hepatocyte proliferation and suppressing their apoptosis by intraportal MSCs transplantation [123]
Bone marrow	Rat	Attenuated aggregation and function of neutrophils [118]
Adipose tissue	Mice	Protection against ALF by affecting the Nrf2 and cytochrome P450 expression [201]
Umbilical cord	Mice	Inducing the endogenous liver regeneration but not notable hepatogenic differentiation [202]
Umbilical cord	Mice	Attenuation of ALF by down-regulation of MyD88/NF-κB pathway involved in inflammation [203]
Bone marrow	Mice	Attenuation of ALF by modifying ratio between Th17 and regulatory NKT cells [204]

Aspartate aminotransferase (AST), Alanine aminotransferase (ALT), Glycogen synthase kinase-3β (GSK-3β), Mammalian target of rapamycin (mTOR), Phosphoinositide 3-kinases (PI3Ks), CXC chemokine receptor 4 (CXCR4), Stromal derived factor-1α (SDF-1α or CXCL12), Prostaglandin E2 (PGE2), Nuclear factor-erythroid factor 2-related factor 2 (Nrf2), Nuclear factor-kappa B (NF-κB), Natural killer T (NKT) cells, T helper 17 (Th17), Interleukin-10 (IL-10)

**Table 2:** Administration of modified mesenchymal stromal cells (MSCs) or/and native MSCs in combination with other modalities in liver failure preclinical models, especially acute liver failure (ALF)

Sources	Model	Intervention	Result (ref)
Adipose tissue	Rat	MSCs plus Eugenol	Enhancing antifibrotic competencies of MSCs by eugenol through down-regulation of TGF-β/Smad axis [205]
Bone marrow	Rat	MSC plus Neutrophil depletion	Amelioration of ALF in rats [206]
Umbilical cord	Rat	MSC plus Icaritin	Enhancing the antiapoptotic capability of MSCs by promoting the HGF/c-Met pathway [131]
Umbilical cord	Mice	HNF4α-overexpressing MSCs plus Hepatocyte	Improving the EGF release by HNF4α-UMSCs [207]
Umbilical cord blood	Rat	VEGF_165_ -overexpressing MSCs	Induction of marked therapeutic influences on ALF [143]
Bone marrow	Mice	CXCR4-overexpressing MSCs	Improved migration and reduced damaged tissue by stimulating hepatoprotective impacts [142]
Amniotic fluid	Rat	IL-1-overexpressing MSCs	Improved liver function along with prolonged survival [145]
NA	Swine	MSCs plus IL-lRa-loaded chitosan nanoparticles	Eliciting a synergistic impact by abrogating liver inflammation [136]
Bone marrow	Rat	Dexmedetomidine and Midazolam primed MSCs	Enhancing the therapeutic merits of MSCs [208]
Umbilical cord	Rat	MSCs plus G-CSF	Attenuation of liver damage by suppressing the generation of pro-inflammatory cytokines, alleviation of oxidative stress, and reducing liver cell loss [132]
Bone marrow	Swine	MSCs plus IL-1R antagonism	Exerting synergistic influences by prohibiting the inflammation and apoptotic signaling [135]
Bone marrow	Mice	MSCs seeded on human amniotic membranes (HAM)	Improving survival rate [209]
Bone marrow	Mice	Poly lactic-co-glycolic acid (PLGA) scaffold loaded with MSCs	Stimulation of hepatoprotective impacts by paracrine factors [127, 128]
Bone marrow	Mice	Regenerated silk fibroin (RSF) scaffold loaded with MSCs	Potentiating liver function by provoking angiogenesis [130]

CXC chemokine receptor 4 (CXCR4), Interleukin-1 (IL-1), Hepatocyte nuclear factor 4 alpha (HNF4α), Transforming growth factor (TGF-β), Vascular endothelial growth factor 165 (VEGF165), Granulocyte colony-stimulating factor (G-CSF), Hepatocyte growth factor (HGF)

### MSCs delivery using biomaterials

Present cell transplantation approaches are hindered via poor post-delivery survival, liver ECM and vasculature deterioration, and also difficulties in fusion into the host tissue [126]. As a result, scientists are persuaded to deliver MSCs within biomaterial structure to sustain the transplants’ viability and also potentiate MSCs long-standing activation in vivo [126].

Recent reports noted that BM-MSCs are valued options to co-culture with hepatocytes in poly (lactic acid-glycolic acid) (PLGA) scaffolds, enhancing the hepatocellular activities [127]. Administration of BM-MSCs and hepatocyte seeded PLGA scaffolds led to the considerably advanced cellular proliferation and conversely supported a striking reduction in ALT, AST, and total bilirubin in ALF preclinical models post-transplantation, ultimately leading to the prolonged survival [127]. Another study demonstrated that MSCs seeded PLGA scaffolds were survived for 3 weeks, and displayed more evident activities than MSCs injected by intravenous route, which was verified by lower mortality in vivo [128]. However, there was no significant alteration in hepatic inflammation and necrosis zones between the two applied interventions [128]. Also, poly L-lactic acid (PLLA) nanofiber scaffold could improve the hepatic differentiation of BM-MSCs [129]. In vitro, analysis exhibited that expression levels of liver-specific markers, more importantly, albumin and α-fetoprotein, were greater in differentiated cells on the nanofibers compared with differentiated cells in plates. These results deliver the proof of the theory that engineered PLLA scaffold could be an efficient alternative to augment MSCs differentiation into functional HLCs [129]. Besides, BM- and AT-MSC seeded regenerated silk fibroin (RSF) matrices potently differentiated into HLCs in vitro and also stimulated angiogenesis and restored liver functions in the ALF mice model in vivo [130].

### Combination therapy with MSCs

A diversity of studies recently has focused on combination therapy with MSCs and other molecules or modalities to diminish ALF. Meanwhile, co-administration of MSCs with Icaritin, a well-known ingredient isolated from traditional Chinese medicine, resulted in promising outcomes in vivo [131]. Indeed, MSCs co-cultured with Icaritin improved survival, reduced serum levels of AST and ALT, and elicited histological variations in liver tissue more potently than MSCs alone. Importantly, the up-regulation of HGF/c-Met by Icaritin was found to be involved in MSCs-triggered antiapoptotic influences on hepatocytes, reflecting the potential of herbal extracts to promote MSC-mediated therapeutic impacts [131]. The addition of the granulocyte colony-stimulating factor (G-CSF) to UCB-MSCs also improved survival and reduced ROS and pro-inflammatory cytokines expressions in ALF murine model [132]. Also, intervention engendered a significant reduction in cell apoptosis in liver tissues more evidently than UCB-MSCs alone [132]. These findings were similar to previous reports displaying that G-CSF therapy alone could significantly attenuate short-term mortality in patients suffering from liver failure mainly by reducing inflammation concomitant with activating PI3K/Akt axis in hepatocytes [133, 134]. In another study, thanks to the crucial role of IL-1 in the progress of ALF, Sang et al. evaluated possible synergetic effects of combined use of MSCs with 2 mg/kg interleukin-1 receptor antagonist (IL-1Ra) in vivo [135]. They found that treatment significantly attenuated liver cell apoptosis, improved their proliferation, and eventually enhanced animal survival. It is supposed that the observed effects were dependent on enhancement in AKT and also a reduction in nuclear factor (NF)-κB expression, potentiating liver cell proliferation [135]. Similarly, co-administration of MSCs plus IL-1Ra chitosan nanoparticles (NPs) was more effective than MSC transplantation alone for treating ALF [136]. IL-1Ra-loaded NPs administration by ear veins exhibited synergistic impacts with portal vein injection of MSC in a mini swine model of ALF by the hindrance of liver inflammation [136].

### Pretreated MSCs

Current studies have verified that pretreatment with chemical agents, hypoxia, and also cytokine or chemokine in vitro can improve the therapeutic merits of MSCs upon transplantation in vivo [137, 138]. Compared to native MSCs, pretreated MSCs largely demonstrate developed hepatogenic differentiation, homing capability, and survival and paracrine impacts.

In 2019, Nie et al. suggested that IL-1β pretreatment could circumvent the MSC's poor migration toward the injured region in ALF murine model [139]. Correspondingly, IL-1β-MSCs showed superiority over native MSCs respecting the survival time and liver function in vivo. Remarkably, IL-1β-MSCs suppressed liver cell apoptosis and necrosis and also provoked their proliferation. Preferred effects were most probably enticed by improved CXCR4 expression resulting from IL-1β pretreatment and thereby increased migration toward CXCL12 (SDF-1 α) in damage tissue [139]. Interestingly, pretreatment with injured liver tissue might improve MSCs homing and also their hepatogenic differentiation [140]. In vivo, transplantation of pretreated MSCs led to an enhancement in albumin, cytokeratin 8, 18, and antiapoptotic protein Bcl-xl levels, whereas supported a reduction in pro-apoptotic protein Bax and caspase-3 levels [140]. Likewise, short-term, but not long-term, sodium butyrate (NaB) treatment augmented hepatogenic differentiation of BM-MSCs and consequently alleviated liver injury in vivo, according to Li et al. reports [141].

### Genetically modified MSCs

Genetically modified MSCs mainly are used to enhance their colonization rate post-transplantation, leading to ameliorated liver recovery in ALF. Meanwhile, genetically modified MSC to overexpress the CXCR4 gene demonstrated more appropriate migration capability toward SDF-1α and also induce better hepatoprotective impacts in vitro [142]. In ALF murine model, CXCR4-MSCs efficiently migrated to damaged tissue, and in turn, brought about prolonged survival and restored liver activity more prominent than native MSCs transplantation [142]. Besides, UCB-MSCs modified to overexpress vascular endothelial growth factor 165 (VEGF165), a strong pro-angiogenic factor, potentiated the UCB-MSCs multipotency and also resulted in better homing and colonization in liver tissues post-transplantation [143]. While both native UCB-MSCs and VEGF_165_-encoding UCB-MSC restored liver activity in the ALF rat model, modified stromal cells exhibited more desired therapeutic influences on ALF [143]. Given that IL-35 plays a pivotal role in Treg-induced immunoregulation, Wang et al. evaluated the therapeutic merits of IL-35 overexpressing MSCs in ALF [144]. They showed that modified stromal cells migrated to the damaged tissues and considerably reduced the necrosis zones of damaged livers. Moreover, IL-35-MSCs averted hepatocyte apoptosis through down-regulation of the FASL expression by immune cells. They also attenuated IFN-γ levels secreted by immune cells potently via targeting JAK1-STAT1/STAT4 signal pathway [144]. As described in previous sections, IL-1Ra elicits strong anti-inflammatory and antiapoptotic impacts on immune response in liver failure. Accordingly, Zheng and coworkers showed that transplantation of IL-1Ra-encoding amniotic fluid (AF)-MSCs by the portal vein in the ALF rat model led to reduced mortality as well as ameliorated liver activity [145].

## MSCs-exosome in ALF

Exosomes are small membrane-bound EVs that are produced and then released by numerous types of cells, such as stem/stromal cells, immune cells, or tumor cells. Exosomes are comprised of a myriad of biological components, including proteins, lipids, mRNAs as well as miRNAs as cargo, which can be conveyed to the recipient cells [103]. Such cargo can adjust physiological cell functions and thereby adapt tissue microenvironment, and also inspire hepatocyte proliferation, reflecting their competencies to be described as a rational therapeutic option in liver diseases, such as ALF (Table 3). Reduced levels of miR-20a-5p accompanied with the enhanced level of CXCL8, most eminent neutrophil chemoattractants, are mainly observed in hepatocytes during ALF. But, BM-MSCs-exosome could improve miR-20a-5p expression and conversely attenuate CXCL8 levels in hepatocytes [146]. Also, systemic injection of UC-MSC-exosome (16 mg/kg) induced liver restoration in the ALF mice model [105]. It was found that glutathione peroxidase1 (GPX1) enriched exosome-mitigated oxidative stress and apoptosis in the hepatocyte, while the elimination of GPX1 led to the abrogated UC-MSCs-exosome-elicited hepatoprotective impacts in mice [105]. In addition, UC-MSC-exosomes potently modified the membranous expression of CD154 (or CD40 ligand) in intrahepatic CD4^+^ T cells, largely contributing to the inflammatory response in the liver [147]. The suppressive effect on CD154 expression and resultant inflammation was due to the existence of chaperonin containing TCP1 subunit 2 (CCT2) in these exosomes, which targets Ca2 + influx and down-regulates CD154 generation in CD4 + T cells [147]. In another study, Shao et al. screened the miRNAs in the MSCs-exosomes complicated in inhibition of IL-6-mediated signaling axis in ALF mice model. They showed that miR-455-3p was released by exosomes and efficiently instigated PI3K signaling, and in turn, sustained hepatocyte proliferation [106]. Also, IL-6 pretreated MSCs or exosomes exhibited higher levels of miR-455-3p compared with native MSCs or their derivative exosome. In fact, miR-455-3p-enriched exosomes suppressed macrophages activation, reduced local liver injury, and also diminish the expression of pro-inflammatory cytokines in vivo [106]. The miR-455-3p also could constrain activation of HSCs and liver fibrosis upon down-regulation of the heat shock protein (HSP) 47/TGF-β/Smad4 signaling pathway [148]. Importantly, C-reactive protein (CRP) enriched placenta-derived mesenchymal stromal cells (PD-MSCs)- exosome could up-regulate Wnt signaling pathway as well as angiogenesis in animal hepatocytes by interacting with endothelial cells [149]. Another study also revealed that rat BM-MSCs-exosome-rich fractionated secretome could bring about a hepatoprotective impact in ALF models mainly caused by diminished oxidative stress [150]. Similarly, transplantation of exosomes derived from menstrual blood-mesenchymal stromal cells (Men-SCs) that contained a diversity of cytokines, such as intercellular adhesion molecule-1 (ICAM-1 or CD54), angiopoietin-2, Axl, angiogenin, insulin-like growth factor-binding protein 6 (IGFBP-6), osteoprotegerin, IL-6, and IL-8, improved liver function in the ALF animal model [151]. Treatment resulted in improved survival rates as well as reserved hepatocyte apoptosis. Notably, attenuated numbers of neutrophils and also diminished levels of caspase-3 were evidenced post-transplantation, assuming that Men-SC-exosome can be a substitute treatment to support liver failure [151]. Pretreatment of UC-MSCs-exosome with TNF-α also enhanced exosome-induced hepatoprotective influence in the ALF mice model [107]. Pretreated exosomes led to the attenuated serum ALT, AST, and pro-inflammatory cytokines levels and concomitantly down-regulated stimulation of NLRP3 inflammasome. Molecular analysis revealed that miRNA-299-3p up-regulated in TNF-α-primed MSCs-exosome played an eminent role in the amelioration of liver damage in ALF by blocking the NLRP3 pathway [107]. Apart from its role in liver failure recovery, a miR-299-3p activity as a potent tumor suppressor has been documented in hepatocellular carcinoma by alleviating tumor size and venous infiltration [152].

**Table 3:** Mesenchymal stromal cells (MSCs) derived molecules (e.g., exosome) in liver failure preclinical models, especially acute liver failure (ALF)

Sources	Model	Intervention	Result (ref)
Umbilical cord	Mice	MSCs-exosome	GPX1 enriched exosomes diminished oxidative stress and also apoptosis [105]
Placenta	Rat	MSCs-exosome	CRP enriched exosome provoked angiogenesis by up-regulation of Wnt signaling axis [149]
Bone marrow	Rat	MSCs-exosome	Stimulation of hepatoprotective impacts by exosome-rich fractionated secretome [150]
Bone marrow	Mice	MSCs-exosome	Suppression of NLRP3 in macrophage and thereby reducing ALF by TNF-ɑ pretreated exosome [107]
Menstrual blood	Mice	MSCs-exosome	Liver function recovery, improved survival rates, and suppressed hepatocellular apoptosis [151]
Umbilical cord	Mice	MSCs-extracellular vesicles	Inhibition of T cell activation in liver tissue following reserve of CD154 expression [147]
Bone marrow	Mice	MSCs-conditioned medium	Promoting hepatocyte proliferation, inhibition of their apoptosis, hindrance of the infiltration of macrophages, improving Th2/Th1 ratio, and enabling hepatic stellate cell (HSC) loss [157]
Bone marrow	Rat	MSCs-conditioned medium	Marked attenuation of panlobular immune cells infiltrates and also hepatocellular apoptosis [210]
ESCs-MSCs	Mice	MSCs-conditioned medium	Supporting hepatocytes growth by VEGF enriched conditioned medium [156]
Bone marrow	Mice	MSCs-exosome	Attenuation of liver inflammation by exosomal miR-20a-5p/intracellular CXCL8 axis [146]
Bone marrow	Rat	MSCs-conditioned medium	Reduced hepatocyte apoptosis [154]
Bone marrow	Rat	MSCs-conditioned medium	Improving the hepatoprotective impacts of the conditioned medium by SMGO potently elicited through inhibition of inflammation and loss of hepatocytes [155]
Amniotic fluid	Mice	MSCs-conditioned medium	Hepatic progenitor-like (HPL)-CM showed superiority over amniotic fluid-MSCs in terms of liver recovery [158]

Silica magnetic graphene oxide (SMGO), NLR family pyrin domain containing 3 (NLRP3), Tumor necrosis factor-ɑ (TNF-ɑ), T helper 1/2 (Th1/2), Vascular endothelial growth factor (VEGF), Interleukin 8 (IL-8 or CXCL8), Conditioned medium (CM), Embryonic stem cells (ESCs), Glutathione peroxidase1 (GPX1), C-reactive protein (CRP)

MSCs-conditioned medium (CM) could also modify morphological characteristics of hepatocytes in the ALF model. Meanwhile, secretome achieved by cultivating MSCs with low oxygen content (10%) provoked more prominent hepatoprotective influence, and significantly reduced ALT and AST and also pro-inflammatory cytokines serum levels following injection in vivo [153]. In another study, Li and coworkers evaluated the therapeutic merits of CM from MSCs co-cultured with hepatocytes in the ALF rat model [154]. The apoptotic cells frequency was lower in CM derived from co-cultured cells than MSCs-CM or hepatocyte-CM. Also, CM derived from co-cultured cells strikingly alleviated liver injury and facilitated liver recovery, indicating the advantages of this strategy for liver failure therapy [154]. Also, silica magnetic graphene oxide (SMGO) could enhance the hypo protective influences of MSC-CM in ALF in vivo [155]. Meanwhile, administration of 300 μg/kg SMGO boosted MSC-CM competencies to avert necrosis, apoptosis, and inflammation of hepatocytes. Besides, SMGO therapy up-regulated the expression of VEGF and matrix metalloproteinase-9 (MMP-9) in vitro [155]. Another report also demonstrated that administration of CM from embryonic stem cell (ESC)-derived MSCs potentiated the proliferation of primary hepatocyte and improved IL-10 secretion from immune cells in vivo [156]. It appeared that such events might arouse because of the existence of VEGF in ESC-MSC-CM, which affect hepatocytes proliferation and migration, generating new avenues to cure ALF [156]. Likewise, MSC-CM sustained hepatocytes proliferation, reduced their apoptosis, compromised macrophages infiltration, elevated Th2 and Treg cells population, decreased levels of Th17 cells population, and eventually enabled HSCs death in ALF preclinical model [157]. The MSC-CM injection caused glycogen synthesis and storage recovery and also ameliorated ALF with no effect on Th1 cells [157]. Also, CM achieved from either amniotic fluid (AF)-MSCs or hepatic progenitor-like (HPL) cells derived from AF-MSCs thanks to the presence of IL-10, IL-1Ra, IL-13, and IL-27 stimulated liver recovery in the mice model with ALF [158].

## MSCs in liver-associated conditions (clinical trials)

**Fig. 2:**
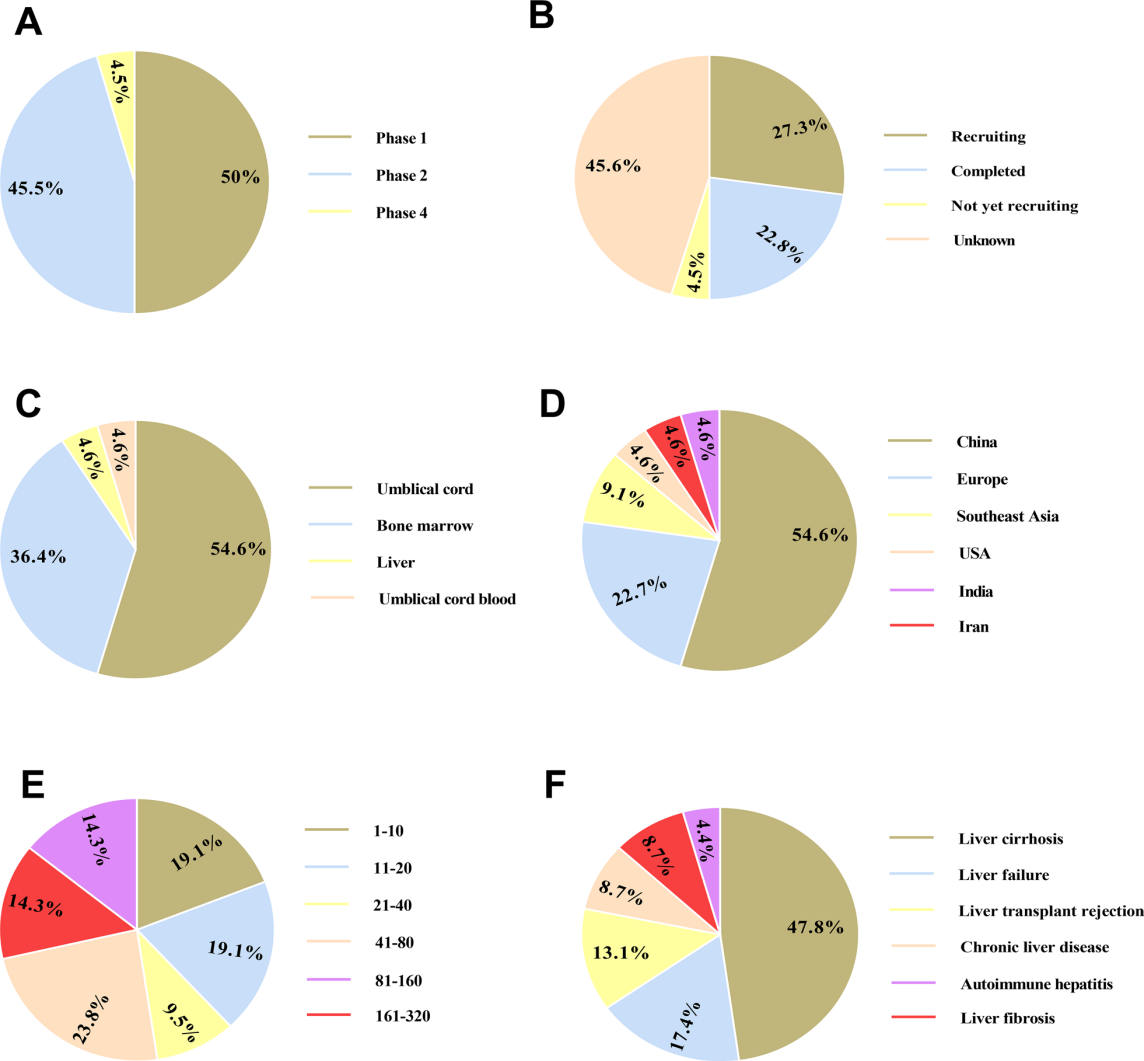
Clinical trials based on mesenchymal stromal cells (MSCs) therapy in liver-associated conditions registered in ClinicalTrials.gov (November 2021). The schematic demonstrates clinical depending on the study phase (**A**), study status (**B**), MSCs source (**C**), study location (**D**), participant number (**E**), and condition (**F**)

Several clinical trials have been accomplished or are ongoing to address the safety, feasibility and efficacy of MSCs therapy in liver-associated conditions, most frequently in liver failure and liver cirrhosis (Table 4, Fig. 2). BM-MSCs and UC-MSCs are most commonly used types of cells. Of course, there is still no definite standard for which source of MSCs should be applied for clinical use. It seems that UC-MSCs are preferred for liver failure treatment as a result of higher differentiation capability. Also, the immunogenicity of UC-MSCs is lower than that of BM-MSCs [159, 160]. Hence, autologous BM-MSCs and allogeneic UC-MSCs are highly preferred. On the other hand, poor proliferation, anti-inflammatory and self-renewal ability impedes AT-MSCs application in clinic [161]. Although intravenous injection is most used route, intraportal administration is evidently the optimal route for MSC therapy in liver-associated conditions due to the faster engraftment and the prohibited off-target accumulation. However, we must assess patients’ conditions and the potential risk of applying a particular route before choosing the administration route.

**Table 4:** Clinical trials based on MSCs-based therapies in liver diseases (e.g., ALF)

Condition	Cell Source	Participant no	Main results (ref)
Primary biliary cirrhosis	Allogeneic UC	7	Robust attenuation in serum ALP and GGT levels [168]
Liver failure	Allogeneic UC	43	Enchantment in the survival rates without side effects [162]
HBV-induced liver cirrhosis	Autologous BM	56	Improving the Treg/Th17 cell ration [171]
Liver cirrhosis	Autologous BM	25	Removing the HCV RNA caused by transplanted MSCs-mediated paracrine effect [211]
Decompensated liver cirrhosis	Autologous BM	4	Improved the quality of life without serious side effects [167]
Alcoholic liver cirrhosis	Autologous BM	12	No side effects in concomitant with histological and quantitative amelioration [212]
HCV-induced liver cirrhosis	Autologous BM	40	Normalization of liver enzymes levels in association with restoration in liver function [213]
Liver cirrhosis	Autologous BM	8	Improved liver function evidenced by enhanced serum albumin and reduced total bilirubin[172]
Liver failure	Autologous BM-derived hepatocyte	40	Improvement in ascites, lower limb edema as well as serum albumin levels [163]
Decompensated liver cirrhosis	Allogeneic UC	45	Improved level function documented with enhanced serum albumin levels and reduced total bilirubin levels [180]
HCV-induced liver cirrhosis	Autologous BM	20	Amelioration of liver function in Egyptian patients [170]
HCV-induced liver cirrhosis	Autologous BM	25	Partial rescue in liver function [169]
Decompensated liver cirrhosis	Autologous BM	27	No significant beneficial effect [174]
Liver failure	Allogeneic BM	110	Improved overall survival and also reduced incidence of severe infections [164]
Liver cirrhosis	Allogeneic (UC, UCB, BM)	26	Stromal cell injection by peripheral vein was safe and partially effective [173]
HBV-induced liver cirrhosis	Allogeneic UC	40	Enhanced IL-10 levels and also reduced IL-6 and TNF-ɑ levels [214]
Ischemic-type biliary lesions following liver transplantation	Allogeneic UC	12	Stem cell injection was safe and elicited favorable short-term outcomes [215]
Alcoholic liver cirrhosis	Autologous BM	72	Ameliorated histologic fibrosis and liver normal activity [216]
Liver allograft rejection	Allogeneic UC	27	Improved Treg/Th17 cell ratio [217]
Liver allograft rejection	Allogeneic BM	10	No side effect [218]

Gamma-glutamyl transferase (GGT), Alkaline phosphatase (ALP), Bone marrow (BM), Umbilical cord (UC), Umbilical cord blood (UCB), Adipose tissue (AT), Hepatitis C virus (HCV), Hepatitis B virus (HBV)

### Liver failure

A study of the safety and preliminary efficacy of UC-MSC transplantation (3 times at 4-week intervals) was carried out by Ming and colleagues [162]. They showed that the intervention had no unwanted effects, while attenuated total bilirubin and ALT levels, prolonged survival rate, and finally ameliorated liver functions, as evidenced by improved serum albumin, and prothrombin activity [162]. As well, intrasplenic and intrahepatic administration of autologous BM-MSCs derived hepatocyte inspired short-term amelioration in patient’s ascites, lower limb edema, and serum albumin [163]. Of course, defining the life span of the transplanted cells, and also determining the presence of long-term side effects is urgently required [163]. Moreover, another trial, which was accomplished from 2010 to 2013, indicated that systemic administration of allogeneic BM-MSCs could exert therapeutic benefits in patients suffering from HBV-related LF [164]. Meanwhile, stromal cell therapy augmented serum total bilirubin and ultimately promoted the 24-week survival rate by stimulating liver rescue concomitant with lessening the occurrences of stern infections compared with the control group (16.1% versus 33.3%) [164]. Likewise, other trials also exhibited that autologous BM-MSC transplantation was safe for chronic HBV-induced LF patients, as shown by the incidence of no serious intervention-related events and carcinoma during 192 weeks follow-up [165]. Also, the short-term outcome was promising; however, long-term efficacy was not clearly amended [165].

### Liver cirrhosis

Cirrhosis is a late-stage liver disease in which healthy liver tissue is substituted with scar tissue and the liver is perpetually damaged. Liver transplantation is a standard therapeutic plan aiming to treat liver cirrhosis patients [166]. Meanwhile, MSCs have recently been noted as a possible therapeutic option to partially ameliorate liver function in this condition as a result of their appreciated antifibrotic and immunoregulatory attributes [55].

A phase 1 trial on 4 patients with decompensated liver cirrhosis verified the safety and feasibility of MSCs therapy [167]. Moreover, the life quality of all patients was ameliorated post-transplantation concerning the mean physical and mental component scales [167]. In primary biliary cirrhosis patients, UC-MSC injection by peripheral vein (3 times at 4-week intervals) exhibited no serious untoward effects [168]. Also, intervention caused a robust reduction in serum alkaline phosphatase (ALP) and γ-glutamyltransferase (GGT) levels compared to the control group during 4 years follow-up. Notwithstanding, no alteration was observed in levels of ALT, AST, total bilirubin, albumin, INR, or the prothrombin time activity. Thereby, comprehensive randomized controlled cohort trials are justified to authorize the clinical merits of UC-MSC transplantation [168]. In addition, injection of autologous BM-MSCs led to a partial amelioration of liver function in 25 Egyptian patients suffering from HCV-triggered liver cirrhosis, as evinced by improved prothrombin activity and serum albumin levels along with reduced bilirubin level [169]. In a similar condition, Amin et al. found that intrasplenic administration of autologous BM-MSCs potentiated liver function with attenuation in total bilirubin, AST, ALT, prothrombin time (PT), and also INR levels [170]. Autologous BM-MSCs therapy also inspired an improvement in liver function among HBV-related liver cirrhosis patients following transplantation [171]. This trial was conducted in 56 patients with HBV-induced liver cirrhosis, and results showed an enhancement in Treg/Th17 ratio post-transplantation during 24-week follow-up [171]. Consistently, mRNA levels of forkhead box protein P3 (FOXP3), an eminent Treg-associated transcription factor, strikingly were diminished, whereas retinoic acid-related orphan receptor gamma t (RORγt) expression levels which are tightly in association with Th17 cells were reduced. Further, the intervention resulted in an enhancement in TGF-β levels, while IL-17, TNF-α, and IL-6 were significantly decreased following transplantation [171]. In contrast to several cited trials implying that the autologous MSC therapy can be a safe and effective alternative for patients with liver cirrhosis [172, 173], Mohamadnejad et al. noted that MSC infusion by peripheral vein had no advantageous result in cirrhotic patients [174]. Overall, large-scale studies are required to achieve reliable results concerning MSCs therapy in liver cirrhosis.

## Potential risks of MSC transplantation

The treatment of liver dysfunctions through MSCs has been the central aim of several clinical and preclinical investigations. In this context, a few issues need to be dealt with cautiously (e.g., the possible emergence of carcinogenesis and the transmission of the virus). Different growth factors can be secreted by MSCs, and this may stimulate the growth of tumor cells and angiogenesis [175, 176]. The past experimental investigations showed that the number of passages is a defining factor in rendering a tissue malignant or cancerous. Studies show that chromosome abnormalities may occur after more than three passages in the MSCs of mice [177, 178]. Moreover, MSCs are likely to experience telomeric deletions after a multitude of passages. Despite the lack of any clinical reports on the malignant transformation of human MSCs, the follow-up period was not long enough for the formation of a tumor for most of them [179]. As a result, there need to be more studies on chromosomal integrity before MSCs transplantation to make sure that the procedure is completely safe.

Contrary to autotransplantation, allotransplantation can pose the danger of the spread of the virus to the patients [180]. Even though the spread of parvovirus B19 into BM cells was observed in vitro, there is no confirmed case of parvovirus B19-positive MSC-related viremia in humans. Yet, we do not know the spread of the herpes simplex virus (HSV) and cytomegalovirus (CMV) via MSCs in vivo. Owing to these facts, recipients, and donors of MSC are recommended to be screened for parvovirus B19, HSV, and CMV, as immunosuppressed patients are likely to catch infectious [181].

## Enhancing the quantity of MSCs-secreted molecules

Now, restricted secretion of soluble mediators, such as exosome, from parental MSCs fences their wide-ranging application in clinics. Following some passages, MSCs mainly demonstrates abrogated competence to produce and then release soluble factor. Recent studies have indicated that tangential flow filtration (TFF) system-based tactics support the secretion of greater levels of vesicles from origin stromal cells than vesicle isolation by ultracentrifuge [182]. Further, ultrasonication of MSC-derived extracellular vesicles could improve their yields up to 20-fold [183]. Other proofs are indicating that three dimensional (3D) culture may facilities the incessant production of MSC-derived exosome [184, 185]. Cultivation of MSCs in 3D cultures together with conventional either differential ultracentrifugation or TFF also could engender a higher quantity of MSCs-derived secretome [186]. Also, MSCs culture on particular biomaterials, such as alginate hydrogel [187] and avitene ultrafoam collagen ease generation of exosome with higher quantity and also potency [188]. As well, pretreatment of MSCs with hypoxia or various molecules, in particular cytokines or chemokines (e.g., IFN-γ, TNFα, IL-1β, IL-6, and TGF-β), gives largely rise to the secretion of vesicles with greater regenerative competencies [189–191].

## Conclusion and future direction

Some investigations in preclinical models of liver diseases, such as ALF, have verified the MSC's unique competence to establish hepatocyte in vivo. Nonetheless, it seems that the therapeutic merits of MSCs largely rely on their aptitudes to secrete a myriad of factors, more importantly, cytokines, growth factors, and miRNAs, facilitating liver recovery. During last two decades, various clinical trials have been conducted to evaluate the capability of MSCs therapy in liver-associated conditions, such as ALF (Table 4, Fig. 2); however, achieved outcomes are quite inconsistent. Given that autologous MSCs derived from elder patients or patients with obesity experienced abrogated proliferation and differentiation capability, using allogeneic cells in some cased is urgently required. In this circumstance, screening recipients and donors of MSC for parvovirus B19, HSV, and CMV are of paramount importance. Taken together, the providing of a universal MSC quality standard evaluation system is required.

To determine the mechanism contributed to MSCs therapy, it is urgently required to determine the protein, DNA and RNA secreted by MSCs. The proteomics and transcriptomics can play a pivotal role in evaluating the underlying mechanism. Notably, improving the frequency of cells homing to the damaged liver is the central point to potentiate the therapeutic impacts of MSCs. In fact, investigation of the homing attributes of MSCs is of paramount importance to augment the effective therapeutic quantity of such cells. In published clinical results, MSCs have been administrated into patients by several available routes, more frequently intravenous routes followed by intrahepatic injection (e.g., by the portal vein and hepatic artery). Also, intrasplenic injection has been applied in a few studies. Based on findings, a remarkable number of cells are trapped in the lungs upon systemic injection and thereby did not move to the liver afterward. Hence, finding better administration route is recommended to achieve significant outcome in vivo. Meanwhile, a study indicated that intraportal injection was more effective than hepatic intra-arterial injection and also intravenous injection to restore liver injury in vivo [123]. As well, it has been shown that portal vein injection has superiority over intrasplenic injection [192]. On the other hand, other reports exhibited that injection by the hepatic artery was not beneficial for the transdifferentiation of MSCs.

Among the recent clinical trials concerning the MSCs therapy for liver diseases (e.g., liver failure) treatment, the total number of MSCs employed was from 10^6^ to 10^9^, irrespective of which method was applied to deliver the dose. The large range of doses applied is difficult to explicate as there are few reports including comparisons of several doses in the same clinical trial. Nonetheless, it seems that as few as 1 × 10^7^ cells can be helpful based on recent published results.

In sum, although clinical trials have evidenced the safety and modest efficacy of short-term application of MSCs, further trials are warranted before MSCs application in clinical to treat ALF and other liver-associated conditions for optimizing administration routes as well as dosses.

## Data Availability

Not applicable.

## Abbreviations

MSCs: Mesenchymal stromal cells
ALF: Acute live failure
HGF: Hepatocyte growth factor
TNF α: Tumor necrosis factor α
IFNγ: Interferon gamma
VEGF: Vascular endothelial growth factor
BM: Bone marrow
UC: Umbilical cord
AT: Adipose tissue
miRs: MicroRNAs
TGFβ: Transforming growth factor β
AST: Aspartate aminotransferase
ALT: Alanine aminotransferase
IL: Interleukin
